# Insights into Chemopreventive Effects of Rosmarinic Acid Against Aflatoxin B1-Induced Genotoxic Effects

**DOI:** 10.3390/foods14122111

**Published:** 2025-06-16

**Authors:** Veronika Furlan, Matjaž Novak, Martina Štampar, Alja Štern, Bojana Žegura, Urban Bren

**Affiliations:** 1 Faculty of Chemistry and Chemical Engineering, University of Maribor, Smetanova 17, SI-2000 Maribor, Slovenia; veronika.furlan@um.si; 2 Department of Genetic Toxicology and Cancer Biology, National Institute of Biology, Večna pot 121, SI-1000 Ljubljana, Sloveniabojana.zegura@nib.si (B.Ž.); 3 Faculty of Mathematics, Natural Sciences and Information Technologies, University of Primorska, Glagoljaška 8, SI-6000 Koper, Slovenia; 4 Institute of Environmental Protection and Sensors, Beloruska Ulica 7, SI-2000 Maribor, Slovenia

**Keywords:** rosmarinic acid, aflatoxin B1, chemopreventive effects, antigenotoxic effects, density functional theory, chemical carcinogen scavenger

## Abstract

In this study, the chemopreventive effects of rosmarinic acid (RA), a major phenolic acid of the plant *Rosmarinus officinalis* L., against the carcinogenic naturally occurring mycotoxin aflatoxin B1 (AFB1) were investigated using both in silico and in vitro approaches. The in silico investigation of the chemical reactions between rosmarinic acid and the carcinogenic metabolite of AFB1, aflatoxin B1 exo-8,9-epoxide (AFBO), was conducted by activation free energies calculations with DFT functionals M11-L and MN12-L, in conjunction with the 6-311++G(d,p) flexible basis set and implicit solvation model density (SMD), according to a newly developed quantum mechanics-based protocol for the evaluation of carcinogen scavenging activity (QM-CSA). Following the computational analyses, the chemoprotective effects of RA were further studied in vitro in human hepatocellular carcinoma HepG2 cells by analyzing its influence on AFB1-induced genotoxicity using a comet assay, γH2AX, and p-H3, while its impact on cell proliferation and cell cycle modulation was assessed using flow cytometry. Our computational results revealed that the activation free energy required for the reaction of RA with AFBO (14.86 kcal/mol) is significantly lower than the activation free energy for the competing reaction of AFBO with guanine (16.88 kcal/mol), which indicates that RA acts as an efficient natural scavenger of AFBO, potentially preventing AFB1-specific DNA adduct formation. The chemoprotective activity of RA was confirmed through in vitro experiments, which demonstrated a statistically significant (*p* < 0.05) reduction in AFB1-induced single- and double-strand breaks in HepG2 cells exposed to a mixture of AFB1 and RA at non-cytotoxic concentrations. In addition, RA reversed the AFB1-induced reduction in cell proliferation.

## 1. Introduction

Rosemary (*Rosmarinus officinalis* L.), a member of the Lamiaceae family, is an evergreen medicinal plant whose leaves are commonly used in the Mediterranean diet as a spicy herb and flavoring agent as well as an ingredient in food supplements. In traditional medicine, rosemary has long been valued for its therapeutic properties, particularly for the prevention and treatment of rheumatoid arthritis, colds, and muscle and joint pain [1,2]. Nowadays, the essential oils and extracts obtained from its flowers and leaves are still widely used to treat minor wounds, rashes, headaches, dyspepsia, and renal colic [2]. This valuable medicinal plant contains various bioactive compounds with diverse pharmacological activities, including antioxidative [3,4], anti-inflammatory [5], antidiabetic [6], and antibacterial [7] activities, which have contributed to its widespread use in traditional and modern medicine. In addition, several in vitro [8,9] and in vivo [10] studies have demonstrated that rosemary extracts exert promising anticarcinogenic activities. One of the key bioactive compounds in rosemary and thyme (*Thymus vulgaris*), rosmarinic acid (RA), an ester of caffeic acid and 3,4-dihydroxyphenyllactic acid, is the major polyphenolic acid found in dried rosemary leaves (up to 10 mg/g) [11]. After absorption, RA is hydrolyzed by colonic bacteria into its two constituent organic acids. Caffeic acid is then further metabolized by O-methylation into ferulic acid or by p-dehydroxylation into m-coumaric acid [12]. The half-life of rosmarinic acid is approximately one hour, and the majority of its metabolites are excreted by the kidneys [13]. Therefore, the utilization of drug delivery systems, such as cyclodextrin, chitosan, and lipid nanoparticles, is recommended to improve the shelf-life, stability, and biocompatibility of rosmarinic acid and its biological activity in vivo [14]. In this way, RA could be successfully applied as an additive in functional food products, incorporated into meat products, oils, milk, beverages, and in cosmetic emulsions [15]. Two catechol moieties of RA serve as hydrogen atom donors, which can neutralize free radicals, resulting in strong antioxidative activity [16]. In addition, RA has been reported to exhibit anti-inflammatory [17], antibacterial (against *Staphylococcus aureus*) [18], and anticarcinogenic activities [19,20]. Specifically, the inhibitory effects of RA on the activity of matrix metalloproteinase 9 (MMP-9), which plays a vital role in cancer progression and the formation of metastases in gastric adenocarcinoma CRL-1739 cells, were reported [19]. Moreover, it was observed that RA exhibits significant cytotoxic effects through inhibiting cell proliferation in a time- and dose-dependent manner in liver cancer HepG2 cells compared to an untreated control [20]. It has also been found to induce apoptosis and cell cycle arrest in the G1 phase by blocking the glycolytic pathway in HepG2 cells, and has demonstrated protective effects against chemically induced DNA damage [21,22,23] and mutagenesis [24]. These studies suggest that RA could be considered as a potential anti-cancer agent in various cancer treatments, and its chemoprotective properties also highlight its potential for safeguarding against foodborne carcinogens.

The relationship between mycotoxins, a group of widely distributed food contaminants, and an increased risk of liver cancer is well-established [25]. Aflatoxin B1 (AFB1), the most carcinogenic naturally occurring mycotoxin, has been associated with hepatocellular carcinoma in humans and animals [26]. AFB1 has been reported to be mutagenic, aneugenic, clastogenic, and to cause epigenetic alterations [26,27]. According to the International Agency for Research on Cancer (IARC), it has been categorized as a human carcinogen (Group 1) since 1993 [28]. AFB1 is a secondary metabolite of the fungi *Aspergillus flavus* and *Aspergillus parasiticus*, which develop on crops, such as corn, peanuts, cottonseed, tree nuts, rice, and spices, stored in conditions with high humidity and temperature [29]. The daily intake of AFB1 in developed countries is estimated to be, on average, in nanograms per day [26], while in developing countries, where corn and peanuts constitute a significant portion of the standard diet, its daily intake is estimated to be in micrograms [29,30]. After AFB1 is consumed, it is metabolized in the liver by cytochrome P450 enzymes (CYP450) into the carcinogenic metabolite AFB1 exo-8,9-epoxide (AFBO). AFBO is a highly reactive, electrophilic metabolite of AFB1, which can spontaneously react with nucleic acids, leading to AFB1-specific DNA adduct formation [29]. Predominantly, AFBO binds with DNA nucleobase guanine and forms pro-mutagenic trans-8,9-dihydro-8-(N7-guanyl)-9-hydroxyaflatoxin (AFB1-N7-Gua) adduct. Its further conversion into the AFB1–formamidopyrimidine (AFB1-FAPy) adduct can result in guanine (G)-thymine (T) transversion mutations in the *TP53* tumor suppressor gene in hepatocellular carcinoma patients who consumed high levels of AFB1 [31]. In addition to DNA-specific AFBO toxicity, AFBO can induce less-specific effects, such as the formation of reactive species capable of oxidizing DNA bases, lipids, and proteins [32].

Although several studies have confirmed the anticarcinogenic activities of RA, its potential chemopreventive effects against genotoxic AFB1 remain unknown. Therefore, the aim of the present work was to investigate the chemoprotective mechanisms of RA against AFB1 both in silico and in vitro. The related molecular mechanisms were explored in silico by calculating the activation free energies (ΔG^‡^) for the chemical reaction between RA and AFBO by applying the density functional theory (DFT) functionals M11-L and MN-12L, in conjunction with the 6-311++G(d,p) flexible basis set and implicit solvation model density (SMD) according to the newly developed QM-CSA protocol. The chemoprotective effects of RA against AFB1-induced cytotoxicity and genotoxicity were further investigated in vitro in human hepatocellular carcinoma HepG2 cells using an MTT assay, comet assay, and flow cytometry. The latter facilitated the simultaneous detection of specific lesions, including DNA double-strand breaks (γH2AX antibodies) indicating clastogenic effects, mitotic cells (% of p-H3-positive cells) reflecting aneugenic effects, cell proliferation (KI67 antibodies), and cell cycle analysis (Hoechst staining) within the same cell population.

## 2. Materials and Methods

### 2.1. In Silico Investigation of Alkylation Reactions Between Rosmarinic Acid and AFBO—The Carcinogenic Metabolite of AFB1

To calculate the activation free energy (∆G^‡^) of the reaction between RA and the carcinogenic metabolite aflatoxin B1 exo-8,9-epoxide (AFBO), the quantum mechanics-based protocol for the evaluation of carcinogen scavenging activity (QM-CSA) was employed [33]. According to the QM-CSA protocol, density functional theory (DFT) M11-L and MN12-L functionals, in conjunction with the 6-311++G(d,p) flexible basis set and implicit solvation model density (SMD) [34] were incorporated. The quantum mechanical calculations of ∆G^‡^ were performed using Gaussian 16 on the HPC RIVR supercomputer Vega located at the Institute of Information Science (IZUM). To evaluate the computational results, the calculated ∆G^‡^ for the reaction between RA and AFBO was compared to the ∆G^‡^ for the reaction between AFBO and guanine, calculated using analogous computational methodology. According to the QM-CSA protocol, the calculated ∆G^‡^ for the reaction between guanine and AFBO using M11-L and MN12-L functionals in combination with the 6-311++G(d,p) flexible basis set and SMD gave the best agreement with the corresponding experimental counterpart, which was obtained based on the transition state theory of Eyring:

(1) $$k=\frac{k_{B}T}{h}e^{\left(-\frac{\Lambda G^{‡}}{k_{B}T}\right)}$$

where *k* represents the experimentally determined rate constant, *h* is the Planck constant, *k_B_* is the Boltzmann constant, and *T* is the absolute temperature [35].

**Figure 1:**
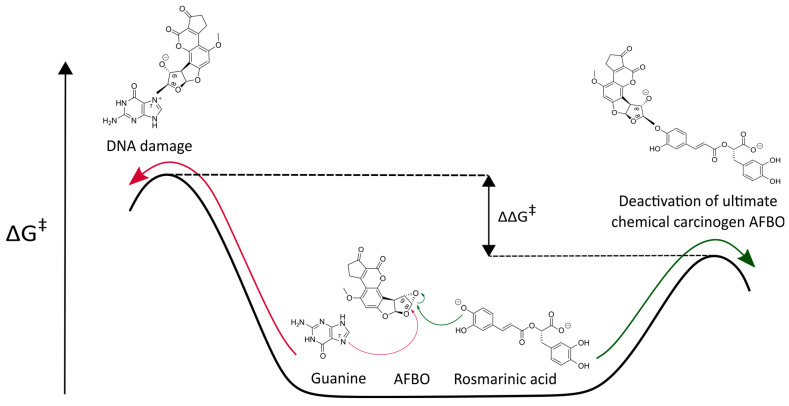
Competing reactions of the carcinogenic metabolite AFB1 exo-8,9-epoxide (AFBO) with rosmarinic acid (green arrow) and guanine (red arrow).

**Figure 2:**
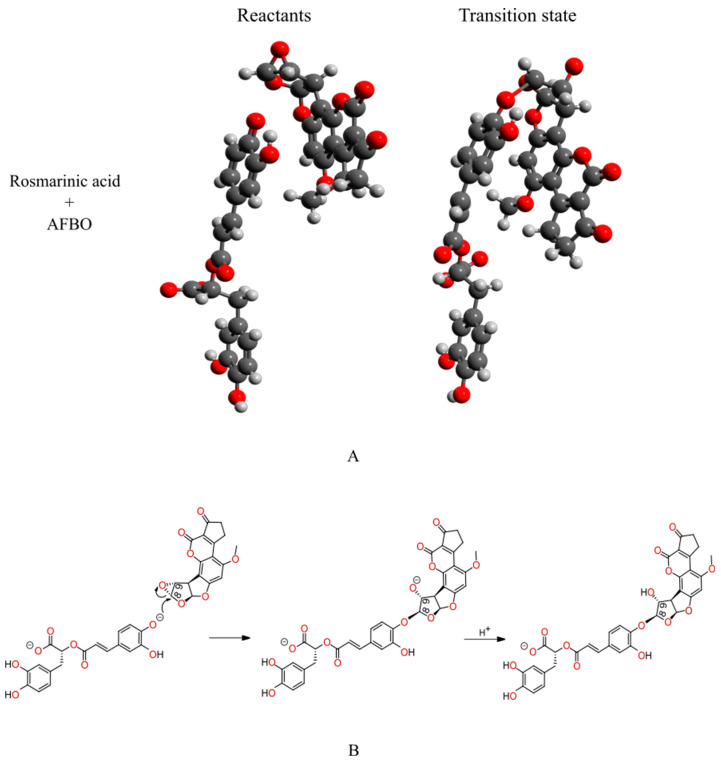
(**A**) The structures of the reactants (left) and the transition state (right) of the AFBO-RA complex, obtained with the M11-L functional in combination with the 6-311++G(d,p) basis set and SMD. From the reactant and transition state geometries, the change in the molecular orientation after bonding is clearly visible. (**B**) The proposed *S_N_2* reaction mechanism for the reaction of RA with AFBO.

The ∆G^‡^ for the investigated alkylation reaction represents the difference in the ∆G^‡^ between the transition state and the reactant state structures. Firstly, the reactant and transition state structures had to be obtained. A geometry optimization and vibrational analysis were performed to confirm the reactant structure, which represents the local minimum on the potential energy surface. The approximate transition state structure was then identified by a relaxed potential surface scan [36]. This structure was then selected as the starting point for the Berny algorithm and frequency analysis, which was used to confirm if the obtained transition state structure was correctly optimized, representing the first-order saddle point on the potential energy surface [37,38]. RA was studied in nucleophilic (di-anionic) form at physiological conditions. The MarvinSketch 20.19.0 software package was applied to predict the pKa values for the most nucleophilic oxygen atoms of RA at pH 7.4 [39]. The ChemDraw 12.0 program was used to depict the proposed *S_N_2* reaction mechanism, presented in Figure 1 and Figure 2B. The optimized reactant and transition state structures, presented in Figure 2A, were visualized in Avogadro [40].

### 2.2. Chemicals

Rosmarinic acid (RA), Aflatoxin B1 (AFB1), sodium pyruvate ethylenediaminetetraacetic acid (EDTA), dimethylsulfoxide (DMSO), Benzo(a)pyrene (BaP), NaHCO_3_, Minimal Essential Medium Eagle (MEM), non-essential amino acids (NEAA), NaCl, NaOH, and 3-(4,5-dimethylthiazol-2-yl)-2,5-diphenyltetrazolium bromide (MTT) were all purchased from Sigma-Aldrich (St. Louis, MO, USA). Phosphate-buffered saline (PBS), L-glutamine, ethanol, penicillin/streptomycin, and fetal bovine serum were obtained from PAA Laboratories (Toronto, ON, Canada). Triton X-100 was procured from Thermo Fisher Scientific (Pittsburgh, PA, USA). Hoechst 33258, trypsin, low-melting-point agarose (LMP), and normal-melting-point agarose (NMP) were purchased from Invitrogen (Waltham, MA, USA). GelRed Nucleic Acid Stain was acquired from Biotium (Fremont, CA, USA). Tris was obtained from Merck (Darmstadt, Germany). Anti-H2AX pS139-APC, anti-Histone H3 pS28-PE, anti-Ki67-FITC, REA Control (I)-FITC, REA Control (I)-PE, and REA Control (I)-APC antibodies were obtained from Miltenyi Biotec (Bergisch Gladbach, Germany). All other reagents were of the purest grade, and solutions were made using Milli-Q water (Millipore Corporation, Darmstadt, Germany). Stock solutions of RA (16.653 mM) and AFB1 (32 mM) for in vitro studies with HepG2 cells were prepared in DMSO and stored at −20 °C.

### 2.3. Human Hepatoma HepG2 Cells

HepG2 cells were obtained from the American Type Culture Collection (HB-8065™, ATCC, Manassas, VA, USA). The cells were cultured at 37 °C in a 5% CO_2_ moisturized atmosphere in MEME medium supplemented with 2.2 g/L NaHCO_3_, 2 mM L-glutamine, 1% NEAA, 1 mM sodium pyruvate, 10% FBS, and 100 IU/mL streptomycin/penicillin.

### 2.4. Determination of Cell Viability Using an MTT Assay

The impact of RA on AFB1, as well as the chemoprotective effects of RA against AFB1-induced cytotoxicity, were determined using an MTT (3-(4,5-dimethylthiazol-2-yl)-2,5-diphenyltetrazolium bromide) reduction assay according to Mosmann [41], with minor modifications [42]. Before the treatment, HepG2 cells were seeded onto a 96-well microplate (Corning Costar Corporation, New York, NY, USA) at a density of 8000 cells/well and incubated overnight at 37 °C to attach. The next day the growth medium was replaced by fresh media containing RA (0.625, 1, 2, 4, 5, 6, 8, and 10 µM), AFB1 (30 µM), or binary mixtures of RA (0.625, 1, 2, 4, 5, 6, 8, and 10 µM) and AFB1 (30 µM). After 24 h incubation, MTT (final concentration of 0.5 mg/mL) was added to the exposed cells and incubated for an additional 3 h. At the end of the incubation, the medium was removed, and formazan crystals were dissolved in DMSO. The optical density (OD) was determined at 570 nm, using 690 nm as the reference wavelength, with a microplate spectrofluorometer (Synergy MX, BioTek, Winooski, VT, USA). For each experiment, negative (cell medium), solvent (0; cells exposed to 0.16% DMSO), and positive (PC; 4% DMSO) controls were included. The cell viability was measured for three independent experiments, each time with 5 replicates per experimental point.

The cell viability was determined by comparing the OD of the treated cells to that of the solvent-treated cells (0.16% DMSO). The differences in cell viability among the treated groups and the solvent control group were analyzed using a one-way analysis of variance (ANOVA) and Dunnett’s multiple comparison test with the program GraphPad Prism V10 (GraphPad Software, San Diego, CA, USA). *p* < 0.05 value was considered statistically significant.

### 2.5. The Comet Assay

A comet assay was performed according to Collins et al. [43], with minor modifications as described by Novak et al. [44]. The cells were seeded onto 12-well tissue-culture-treated plates (Corning Costar Corporation, New York, USA) at a density of 80,000 cells/well and incubated for 24 h to attach. Following incubation, the growth medium was replaced with fresh medium containing either RA (0.008, 0.04, 0.2, 1, and 5 µM), AFB1 (30 µM), or binary mixtures of RA (0.3125, 0.625, 1.25, 2.5, and 5 µM) and AFB1 (30 µM). After exposure, the HepG2 cells were trypsinized, collected, and centrifuged. A 30 μL cell suspension was mixed with 70 μL of 1% LMP agarose and layered onto fully frosted slides pre-coated with 1% NMP agarose. The cells were lysed (2.5 M NaCl, 100 mM EDTA, 10 mM Tris, 1% Triton X-100, and pH 10) for 1 h at 4 °C, followed by DNA denaturation in an electrophoresis buffer (1 mM EDTA, 300 mM NaOH, and pH 13) for 20 min at 4 °C. Electrophoresis was performed at 25 V (1 V/cm) for 20 min. The staining of the nuclei was performed using GelRed (Biotium, Fremont, CA, USA). For each experimental condition, the images of 50 randomly selected nuclei were analyzed across three independent experiments using a fluorescence microscope (Nikon, Eclipse 800, Tokyo, Japan) at 400× magnification. The imaging analysis was conducted with Comet Assay IV software (Perceptive Instruments Ltd., Suffolk, UK). Benzo(a)pyrene (BaP; 30 μM) was applied as the positive control. In each experiment, a solvent control (0; growth medium containing 0.036 *v*/*v* % of DMSO) and a negative control (growth medium) were included. The results were expressed as the % of the tail DNA. Statistical analysis was performed using a one-way analysis of variance (non-parametric ANOVA, Kruskal–Wallis test) to test the differences in the % of tail DNA between the treated groups and the solvent control.

### 2.6. Flow Cytometric Analyses of Gamma-H2AX Formation, Histone p-H3-Positive Cells, Cell Proliferation, and Cell Cycle

The induction of DNA double-strand breaks (DSBs; γH2AX antibody), the formation of mitotic cells (p-H3 antibody), cell proliferation (Ki67 antibody), and cell cycle distribution (Hoechst staining) were analyzed using flow cytometry, according to Štampar et al. [45], with minor modifications. The cells were seeded onto T-25 tissue-culture-treated plates (Corning Costar Corporation, New York, USA) at a density of 450,000 cells/well and incubated overnight to attach. The growth medium was then replaced with fresh medium containing RA (0.625, 1.25, 2.5, and 5 µM), AFB1 (30 µM), or binary mixtures of RA (0.625, 1.25, 2.5, and 5 µM) and AFB1 (30 µM). After 24 h of exposure, the cells were first fixed in 4% PFA for 15 min, washed in cold PBS, and stored in 0.5 mL PBS at 4 °C until analysis. After fixation, the cells were rinsed with cold PBS and subsequently labeled to enable the simultaneous detection of four distinct endpoints. First, they were labeled with antibodies anti-H2AX pS139-APC, anti-Histone H3 pS28-PE, and anti-Ki67-FITC (50-fold diluted antibodies in 1% BSA containing 0.1% Triton X-100) for 30 min, at room temperature, in the dark, washed with 1% BSA, and subsequently stained with Hoechst 33342 nuclei dye (diluted in 0.1% Triton X-100 1:1000) for 20 min at room temperature and in the dark, as described by Štampar et al. [46].

Flow cytometric analysis was performed using MACSQuant Analyzer 10 (Miltenyi Biotec, Germany). To minimize non-specific antibody binding, Rea-FITC, Rea-APC, and Rea-H3 internal controls (Miltenyi Biotec, Germany) were employed. Etoposide (1.7 μM) and colchicine (0.1 μM) were included as positive controls (PC). Each experimental condition was assessed in three independent biological replicates, with 20.000 single-cells events recorded per sample. Data analysis was conducted using FlowJo software version 10 (New York, NY, USA). Visualization and statistical analysis were performed with GraphPad Prism software V10 (GraphPad Software, San Diego, CA, USA). The difference in H2AX-positive cells between treated and control groups was evaluated using nested one-way ANOVA (uncorrected Fisher’s LSD), with *p* < 0.05 considered statistically significant. Differences in mitotic cells (p-H3 positive) were assessed by one-way ANOVA with multiple comparisons, with *p* < 0.05 deemed significant. To analyze differences in Ki67-positive cells, an unpaired *t*-test with Mann–Whitney correction was applied, with *p* < 0.05 considered statistically significant. Additionally, cell cycle frequency distributions were investigated using a chi-square test, where *p* < 0.05 was considered statistically significant.

## 3. Results and Discussion

### 3.1. Mechanistic Insights into the Alkylation Reaction Between Rosmarinic Acid and AFBO—The Carcinogenic Metabolite of AFB1

The kinetics of AFBO-RA binding, as well as AFBO–guanine adduct formation, were evaluated by the calculation of the ∆G^‡^. The calculated ∆G^‡^ required for the reaction between RA and AFBO was compared to the ∆G^‡^ of the reaction of AFBO with guanine. A lower ∆G^‡^ corresponds to a faster reaction and signifies preferential binding with the carcinogenic metabolite AFBO. If the ∆G^‡^ of the reaction between RA and AFBO is lower than the ∆G^‡^ of the reaction between guanine and AFBO, then RA is an efficient scavenger of AFBO (Figure 1).

From the structures of the reactants and transition state depicted in Figure 2A, it can be observed that the most nucleophilic phenolic oxygen of RA covalently binds with the least-hindered epoxy carbon atom of AFBO while the epoxy ring is open. The alkylation reaction between RA and AFBO, therefore, corresponds to the *S_N_2* reaction mechanism (Figure 2B).

The calculated ∆G^‡^, with the M11-L and MN12-L functionals in combination with the 6-311++G(d,p) flexible basis set and SMD for the alkylation reaction between RA and AFBO, the lowest real frequencies of the reactant structures, the imaginary frequencies of the transition state structures, and the distances between the reactive centers of the reactants and transition states are summarized in Table S1. The correct reactant state structure must correspond to the local minimum with only real frequencies. The correctly optimized transition state must have exactly one imaginary frequency corresponding to a new covalent bond connecting the nonchiral epoxy carbon of AFBO with the most reactive phenolic oxygen atom of RA, while the epoxy ring of the carcinogenic metabolite AFBO is open. The obtained normal modes (Table S1) correspond to the reaction coordinate, representing the covalent bonding between the most nucleophilic phenolic oxygen of RA and the nonchiral carbon in the epoxy ring of AFBO. In Figure 2B, the proposed *S_N_2* mechanism for the AFBO-RA complex formation is presented.

According to the QM-CSA protocol, the DFT functionals M11-L and MN12-L, in conjunction with the 6-311++G(d,p) flexible basis set and implicit SMD, most accurately reproduced the experimental ∆G^‡^ for the alkylation reactions between guanine and nine carcinogenic metabolites, including AFBO [33]. The functionals M11-L and MN12-L were, therefore, utilized to calculate the corresponding ∆G^‡^ for the reaction between RA and AFBO, and compared with the analogously calculated ∆G^‡^ for the reaction of AFBO with guanine, the most nucleophilic DNA base. The calculated ∆G^‡^ for the reaction between AFBO and guanine was compared with the experimental ∆G^‡^ of the AFBO reaction with guanine in order to determine the combination of the DFT functional, namely M11-L or MN12-L, the 6-311++G(d,p) flexible basis set, and the solvation model SMD, which provided the best agreement with the experimental data.

In Table 1, the results obtained with the M11-L and MN12-L functionals in combination with the 6-311++G(d,p) flexible basis set and the SMD for the reactions of RA and guanine with AFBO are summarized. The experimentally obtained activation free energy for the reaction of AFBO with guanine is also provided [37,38].

According to the QM-CSA protocol, the ∆G^‡^ calculated with the DFT functionals M11-L and MN12-L in combination with the 6-311++G(d,p) flexible basis set and the SMD most accurately reproduced the experimental value for the reaction of AFBO with guanine and, therefore, a very good agreement with the experimental activation free energy for the reaction of AFBO with RA is also expected [33]. From Table 1, it can be observed that the absolute differences between the ∆G^‡^ calculated theoretically using M11-L/SMD and MN12-L/SMD and the experimental value for the reaction between AFBO and guanine are 1.89 kcal/mol and 1.78 kcal/mol, respectively, which represents an acceptable computational error of less than 2 kcal/mol. According to the calculated ∆G^‡^, the same agreement could also be expected for the reaction between AFBO and RA. Therefore, both the M11-L/SMD and MN12-L/SMD methods were chosen to evaluate the reaction of AFBO with RA.

From Table 1, it can be observed that the quantum mechanical calculations, with both the M11-L and MN12-L functionals in combination with 6-311++G(d,p) and the SMD, predicted a significantly higher activation barrier (>1 kcal/mol) for the reaction of AFBO with RA than for its reaction with the most reactive DNA base guanine (by 1.86 kcal/mol and 2.02 kcal/mol, respectively). It can be assumed that RA reacts with AFBO faster than AFBO reacts with guanine, indicating that RA can efficiently prevent the formation of AFBO-DNA adducts. RA, therefore, is a potential primary scavenger of AFBO. Our findings reveal a novel possible mechanism of the cancer-preventing effects of RA against AFBO, which, as a potential scavenger of AFBO, can prevent DNA adduct formation and DNA damage induction. In the following subsections, the chemoprotective effects of RA against AFB1 are further evaluated in vitro by cytotoxicity and genotoxicity assessments, as well as by a cell cycle analysis.

### 3.2. Cytotoxicity of RA, AFB1, and Their Binary Mixtures

In order to assess the protective effects of RA against AFB1-induced cytotoxicity, the effects of RA, AFB1, and their binary mixtures on the viability of hepatocellular carcinoma HepG2 cells were evaluated in vitro by using an MTT assay, a colorimetric assay that measures the reduction of MTT to insoluble formazan by the dehydrogenases in metabolically active HepG2 cells. Assuming that all the tested cell populations have the same metabolic activity, the amount of produced formazan is directly proportional to the number of viable cells.

The results showed that RA (0.625–10 µM) decreased cell viability in a dose-dependent manner after 24 h, reaching 63.59 ± 7.5% at the highest concentration (10 µM). However, at concentrations up to 5 µM, RA did not reduce the cell viability by more than 30% (Figure S1), which is the threshold value typically used for genotoxicity assessment [47]. These findings align with previously published data. Jin et al. [48] reported a similar cytotoxicity of RA on HepG2 cells using a CCK-8 assay after 24 h exposure, with an IC_50_ value of 14 µM and approximately 70% viability at 10 µM. In contrast, several other studies using different viability assays (EZ-Cytox cell viability assay, MTT, and neutral red uptake) have shown much lower cytotoxicity of RA on HepG2 cells after 24 h exposure [49,50,51].

**Figure 3:**
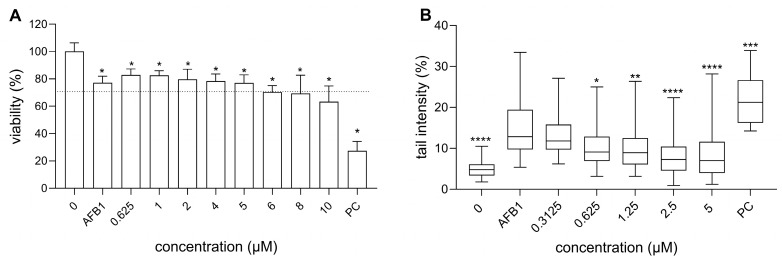
Protective effect of RA against AFB1-induced cytotoxicity (**A**) and DNA strand breaks (**B**) after 24 h exposure. (**A**) The effect of AFB1 (30 µM) and RA combinations on the viability of HepG2 cells, presented as a percentage of the solvent control (0). The cell viability was measured in three independent experiments, each time with 5 replicates per experimental point. A positive control (PC; DMSO 4%) was included in the experiment. The dashed line represents 70% survival with respect to the solvent control. * denotes a significant difference in comparison with 0 (* *p* < 0.05). (**B**) The effect of the AFB1 (30 µM) and RA combinations on DNA damage induction. The data are expressed as the % of DNA in the comet tail and presented as quantile box plots (95% confidence interval), with the mean value in the form of a solid line through the box. The experiments were repeated three times independently, each time analyzing 50 randomly selected nuclei. BaP 30 µM was considered as the PC, and 0 as the solvent control. There were no differences observed between the solvent control and growth medium control. * denotes a statistically significant difference between the AFB1 and the exposed cells (Kruskal–Wallis nonparametric test and Dunn’s multiple comparison test) (* *p* ≤ 0.05, ** *p* ≤ 0.01, *** *p* ≤ 0.001, **** *p* ≤ 0.0001).

In the next step, we investigated the cytotoxic activity of AFB1, both individually and in combination with RA. AFB1 (30 µM) alone reduced cell viability to 77 ± 4.74% (Figure 3A). When combined with RA, the AFB1-induced cytotoxicity remained similar to that of AFB1 alone, suggesting that RA does not significantly alter the cytotoxic effects of AFB1 on HepG2 cells. In contrast, Renzulli et al. [50] reported that the pre-treatment of HepG2 cells with RA (25 and 50 µM) for 24 h induced a clear, dose-dependent protective effect against AFB1-induced cell mortality (2.5, 5, and 10 µM; exposure time, 48 h). In addition, RA exhibited a dose-dependent protective effect against cytotoxicity induced by various genotoxic stressors, such as doxorubicin [52], UVA [53], and t-butyl hydroperoxide [12], in Chinese hamster ovary cells (CHO-K1), human keratocytes, and HepG2 cells, respectively. The authors hypothesized that this protective effect is mainly attributed to the ability of RA to reduce oxidative stress. However, it is important to note that much higher RA concentrations were used in these studies compared to those in our study.

### 3.3. Genotoxicity of RA and AFB1, as Well as RA’s Chemoprotective Effects Against AFB1-Induced Genotoxicity

In order to evaluate the chemoprotective effects of RA against AFB1-induced DNA damage, the genotoxicity of RA was evaluated using an alkaline comet assay, as well as by the detection of H2Ax induction and p-H3-positive cells using flow cytometry. For the genotoxicity assessment, 5 µM of RA was the highest concentration considered non-cytotoxic as evaluated with MTT, and the results showed that RA up to this concentration did not induce DNA damage (Figure 3A). In a previous study, we showed that 30 µM AFB1 induces DNA damage in HepG2 cells after 24 h exposure and reduces cell viability by no more than 30% [38]. Therefore, this concentration was used in subsequent tests to investigate the chemoprotective effect of RA. We demonstrated that RA exhibits a protective effect against AFB1-induced DNA strand breaks in a dose-dependent manner, as evaluated by a comet assay (Figure 3B). The available studies have shown that RA, at non-cytotoxic concentrations, can protect different cell types against DNA strand breaks induced by various genotoxins, such as doxorubicin (chemotherapeutic agent) [23,52], hydrogen peroxide [22], tert-butyl hydroperoxide [54], and UV radiation [53,55,56] after short (1 and 4 h) or long exposure times (24 h) in various experimental set-ups (pre- or co-treatment). Its antigenotoxic potential, in terms of reducing DNA strand breaks induced by tert-butyl hydroperoxide, was found to be lower than that of some other plant phenolic compounds, such as quercetin and luteolin [54]. RA also exhibited activity in vivo (in mice), as it significantly reduced the level of DNA damage induced by ethanol [57], while RA itself did not induce DNA damage in mice and rats [57,58]. This is consistent with the previously published data, which have shown that RA does not induce DNA damage in human keratinocytes (HaCaT) or Chinese hamster lung fibroblasts (V79) after 24 h exposure, even at much higher non-cytotoxic concentrations of 25 and 1120 µM, respectively [23,55].

**Figure 4:**
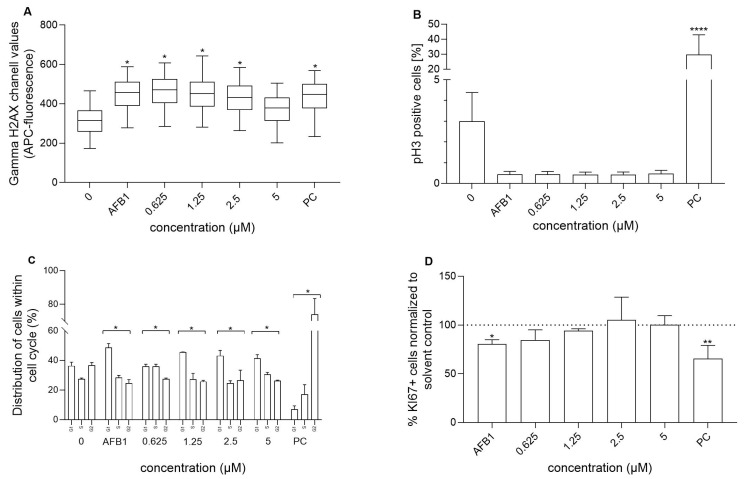
Protective effect of RA against AFB1-induced (30 µM) DNA double-strand breaks (**A**), alterations in the phosphorylation of the histone H3 (p-H3) (**B**), distribution of cells among the phases of the cell cycle (**C**), and the percentage of Ki67-positive cells (**D**) in HepG2 cells after 24 h exposure to AFB1 (30 µM) alone and in combination with RA of different concentrations. The experiments were conducted in three biological replicates; in each sample, 20.000 single cells were recorded. A solvent control (0) and positive control (PC; colchicine 0.1 μM for the phosphorylation of the H3 histone and etoposide 1 µg/mL for the remaining three plots) were included in the experiment. * represents a significant difference in comparison with the solvent control (* *p* ≤ 0.05, ** *p* ≤ 0.01, **** *p* ≤ 0.0001).

Among DNA lesions, DNA double-strand breaks (DSBs) are considered the most harmful, as they can lead to genetic mutations and contribute to the initiation and progression of cancer if not repaired [59]. An early key event in the cellular response to DNA DSBs is the phosphorylation of histone H2AX at the Ser-139 residue (γH2AX), which signals the recruitment and activation of DNA repair proteins to initiate repair. This phosphorylation occurs rapidly, abundantly, and correlates strongly with the number of induced DSBs, as each γH2AX focus represents a single DSB. Therefore, γH2AX is widely recognized as a highly sensitive marker for the detection of DSBs (reviewed in [60,61,62]). We show that RA reduced DNA DSBs induced by AFB1 in a dose-dependent manner, where the DNA DSBs induced by AFB1 were completely reduced at an RA concentration of 5 µM and reached that of the control level (Figure 4A). It has been reported that RA can protect immortalized mouse myoblast cells, primary human neurons, and CHO-K1 cells from DSBs induced by hydrogen peroxide, an oxidative stress trigger; ciguatoxin, a genotoxic compound produced by microalgae; and irradiation, as shown by Western blot, fluorimetry, and immunofluorescence [22,56,63]. The observed protective effect occurred at much higher concentrations than the ones used in our study (50–550 µM). We also show that RA alone did not induce DSBs at non-cytotoxic concentrations (Figure S2A) and that AFB1 significantly induced the formation of DNA DSBs.

The next endpoint assessed in relation to AFB1’s genotoxic activity was the measurement of the percentage of p-H3 positive cells. While γH2AX is a marker of clastogenesis, p-H3 serves as an indicator of aneugensis. Aneugens primarily target non-DNA targets, such as spindle kinases or fibers, disrupting key processes in cell division and leading to defective chromosome segregation. Histone H3 serves as a biomarker for mitotic cells, as it undergoes phosphorylation at serine 10 by the Aurora kinase family during mitosis, facilitating chromosome condensation and segregation [64]. In our study, AFB1 significantly decreased the percentage of p-H3-positive cells compared to the control cells (Figure 4B), and the effect was not reversed/mitigated by RA. Khoury et al. [65] showed that certain clastogens increase γH2AX and decrease p-H3 signalling at high concentrations. Similarly, Sharma et al. [66] demonstrated that histone H3 expression decreases in response to DNA damage in the G1 phase after irradiation of human cells. The authors hypothesized that this phenomenon might be due to global chromatin compaction that represses transcription to facilitate DNA repair and mitotic delay, or it could serve as a trigger to induce a transcription repressive state to facilitate repair. RA alone did not affect the percentage of p-H3-positive cells compared to the control cells (Figure S2B).

### 3.4. Influence of RA and Its Combination with AFB1 on Cell Cycle and Cell Proliferation

To investigate the effect of RA on AFB1-induced cell cycle arrest, the impact of RA, AFB1, and their combination on the cell cycle was assessed. Cell cycle arrest is one of the key cellular responses to DNA damage, allowing cells to repair the damage. If the damage is irreparable, the cell undergoes cell death. Cell cycle delay is a cellular strategy to manage DNA damage or to activate an apoptosis-like program. Our results show that AFB1 indeed induced significant alterations in the cell cycle (Figure 4C), leading to cell cycle arrest in the G0/G1 phase and a concomitant reduction in the proportion of cells in the G2/M phase. A similar effect has been reported in [67,68].

Our findings show that RA alone did not affect the cell cycle of exposed HepG2 cells at the concentrations tested (Figure S2C). Similarly, RA at non-cytotoxic concentrations (5–50 µM) was previously reported not to affect the cell cycle of primary human lung fibroblasts (HLFs), gastric cancer (SGC-7901), breast cancer (MCF-7), or CCR-CEM [69,70,71]. However, at 50 µM, RA slightly but significantly reduced the proportion of HepG2 cells in the G2/M phase after 48 h exposure, with cell viability of 72.34% [70].

While previous studies have shown that RA at concentrations relevant to our study does not markedly influence the cell cycle, we further examined its effects in combination with AFB1. When combined with AFB1, RA did not alter the distribution of cells within the cell cycle, showing no effect on AFB1-induced cell cycle alterations.

Furthermore, we assessed the impact of RA, AFB1, and their combination on the expression of the proliferation marker Ki67 in the same cell population as already analyzed for the cell cycle, p-H3, and γH2AX. Ki67 serves as a biomarker of cell proliferation, as it is expressed in all phases of the cell cycle except G0, thus serving as an indicator of actively dividing cells [72]. Our results show that AFB1 statistically significantly reduced the percentage of Ki67-positive cells (Figure 4D), which aligns with its induction of cell cycle arrest in the G0/G1 phase. Notably, RA was able to reverse this effect in a dose-dependent manner, with a 1.25 µM RA treatment restoring cell proliferation to levels similar to those observed in the solvent-only treated cells. In our study, RA slightly decreased the percentage of Ki67-positive cells, though this decrease was not statistically significant (Figure S2D). This is consistent with previous studies, which have shown that RA can reduce Ki67 expression, as observed in the human hepatocellular carcinoma cell line (SMMC-7721) [73], dissected mouse tumors [74], human osteosarcoma cell lines U2OS and MG63 [75], and in dissected tumor tissues from xenograft nude mice [76].

All in all, the results from our study suggest that RA likely mitigates the effects of AFB1-induced genotoxicity and cell cycle arrest through molecular interactions, probably involving the scavenging of reactive metabolites, such as AFBO, thereby preventing DNA adduct formation and alleviating the impact of AFB1. Taken together, these findings support the potential of RA to modulate the distribution of cells within the cell cycle and to protect them from AFB1-induced toxicity.

## 4. Conclusions

In the present study, the chemoprotective effects of RA and the potential underlying mechanism of its action against the carcinogenic food contaminant AFB1 were investigated, both in silico and in vitro. The computational results reveal that RA is an efficient scavenger of aflatoxin B1 exo-8,9-epoxide (AFBO), the carcinogenic metabolite of AFB1, suggesting its potential to prevent the initiation of chemical carcinogenesis triggered by AFBO. This study also highlights the validity of the proposed *S_N_2* reaction mechanism and demonstrates the applicability of the newly developed QM-CSA protocol to the investigation of reactions between polyphenolic scavengers and epoxy-type chemical carcinogens. The in vitro results further confirm that RA efficiently protected metabolically competent HepG2 hepatocellular carcinoma cells against AFB1-induced DNA strand breaks, supporting its potential chemoprotective role. Overall, this research provides novel insights into the chemoprotective properties of RA and the possible underlying mechanism of its action against the carcinogenic mycotoxin AFB1. Given the widespread occurrence of AFB1 in contaminated food, the chemoprotective effects of RA could have significant implications for mitigating the health risks associated with chronic AFB1 exposure and represent a potential dietary intervention strategy to reduce cancer risk. However, further in-depth in silico and in vitro studies are needed to uncover the precise mechanisms underlying its effects, as well as in vivo studies to confirm its anti-cancer effects and to better validate its role in preventing AFB1-induced carcinogenesis.

## Figures and Tables

**Table 1:** Activation free energies obtained with M11-L and MN12-L functionals in combination with 6-311++G(d,p) basis set and SMD implicit solvation model for reactions of AFBO with RA and guanine.

Scavenger of AFBO	Activation Free Energies		
	$\Delta G_{M11-L/SMD}^{‡}$ ^a^ [kcal/mol]	$\Delta G_{MN12-L/SMD}^{‡}$ ^b^ [kcal/mol]	$\Delta G_{exp}\;*$ [kcal/mol]
RA	15.13	14.86	15.1 [33]
Guanine	16.99	16.88	

^a^ Activation free energy calculated with the M11-L functional and the solvation model SMD. ^b^ Activation free energy calculated with the MN12-L functional and the solvation model SMD. * Experimental activation free energy for the alkylation reaction of AFBO with guanine. According to the QM-CSA protocol, the experimental value serves as a reference to determine the best combination of the DFT functional, basis set, and solvation model for the in silico calculation of the activation free energy for the reaction of AFBO with guanine.

## Data Availability

The original contributions presented in this study are included in the article/Supplementary Material. Further inquiries can be directed to the corresponding author.

## Supplementary Materials

The following supporting information can be downloaded at: https://www.mdpi.com/article/10.3390/foods14122111/s1, Table S1: The obtained results for the reaction of AFBO with rosmarinic acid, applying the M11-L and MN12-L functionals in conjunction with the 6-311++G(d,p) flexible basis set and the implicit solvation model density (SMD); Figure S1: The cytotoxicity and genotoxicity of RA after 24 h exposure. (A) The effect of RA on the viability of HepG2 cells, presented as a percentage of the solvent control (0). A positive control (PC; DMSO 4%) was included in the experiment. The dashed line represents 70% survival with respect to the solvent control. * denotes a significant difference in the comparison with 0 (* *p* < 0.05). (B) The effect of RA on DNA damage induction. The data are expressed as the % of DNA in the comet tail and presented as quantile box plots (95% confidence interval) with the mean value in the form of a solid line through the box. BaP 30 µM was used as the PC and 0 as the solvent control. There were no observed differences between the solvent control and growth medium control. * denotes a statistically significant difference between the AFB1 and the exposed cells (Kruskal–Wallis nonparametric test and Dunn’s multiple comparison test) (**** *p* ≤ 0.0001); Figure S2: The potential of RA to induce DNA double-strand breaks (A), alterations in the phosphorylation of the histone H3 (p-H3) (B), distribution of cells among the phases of the cell cycle (C), and the percentage of Ki67-positive cells (D) in HepG2 cells after 24 h exposure to RA of different concentrations. A solvent control (0) and positive control (PC; colchicine 0.1 μM for the phosphorylation of the H3 histone and etoposide 1 µg/mL for the remaining three plots) were included in the experiment. * denotes a significant difference in comparison with the solvent control (* *p* ≤ 0.05, ** *p* ≤ 0.01, **** *p* ≤ 0.0001).
