# Supplementary material for: Insights into Chemopreventive Effects of Rosmarinic Acid Against Aflatoxin B1-Induced Genotoxic Effects
Source: Foods. 2025 Jun 16;14(12):2111. doi: 10.3390/foods14122111 (PMC12191861; doi:10.3390/foods14122111)
Supplement: Supplementary file 1 [file foods-14-02111-s001.zip › foods-3682428-supplementary.pdf]

Supplementary Materials

Insights into Chemoprotective Effects of Rosmarinic Acid against Aflatoxin B1-Induced Genotoxic Effects

Veronika Furlan[1], Matjaž Novak[2], Martina Štampar[2], Alja Štern[2], Bojana Žegura[2], Urban Bren[1,3,4,*]

[1]Faculty of Chemistry and Chemical Engineering, University of Maribor, Smetanova 17, SI-2000 Maribor, Slovenia
[2]Department of Genetic Toxicology and Cancer Biology, National Institute of Biology, Večna pot 121, Ljubljana 1000, Slovenia
[3]Faculty of Mathematics, Natural Sciences and Information Technologies, University of Primorska, Glagoljaška 8, SI-6000 Koper, Slovenia
[4]Institute of Environmental Protection and Sensors, Beloruska Ulica 7, SI-2000 Maribor, Slovenia

The calculated activation free energies at the functionals M11-L and MN12-L in conjunction with 6-311++G(d,p) flexible basis set for the alkylation reaction of rosmarinic acid with ultimate chemical carcinogen aflatoxin B1 exo-8,9-epoxide (AFBO) solvated with Solvation Model Density (SMD), imaginary frequency of transition state, lowest vibrational frequency of reactant state and the corresponding distances between the reactive centers are collected in Table S1.

Table S1. The obtained results for the reaction of AFBO with rosmarinic acid by applying M11-L and MN12-L functionals in conjunction with 6-311++G(d,p) flexible basis set and SMD implicit solvation model.

Functional /solvation model	$\Delta G^{\ddagger}_{SMD}$ [kcal/mol][a]	ω^{TS} [i cm^{-1}][b]	ω^{R} [cm^{-1}][c]	d^{TS} [Å][d]	d^{R} [Å][e]
**Rosmarinic acid - AFBO**					
M11-L/6-311++G(d,p)	15.13	139.27	10.07	2.07	4.55
MN12-L/6-311++G(d,p)	14.86	141.89	9.82	2.18	4.23

[a] Activation free energies obtained with the functionals M11-L and MN12-L in combination with 6-311++G(d,p) flexible basis set and SMD solvation model. [b] The exactly one imaginary vibrational frequency of the transition state structure. [c] The lowest vibrational frequency of the reactant state structure. [d] The distance in the transition state structure between the most nucleophilic phenolic oxygen of rosmarinic acid, and the achiral electrophilic epoxy carbon of AFBO. [e] The distance in the reactant structure between the most nucleophilic phenolic oxygen of rosmarinic acid and the achiral electrophilic epoxy carbon of AFBO.

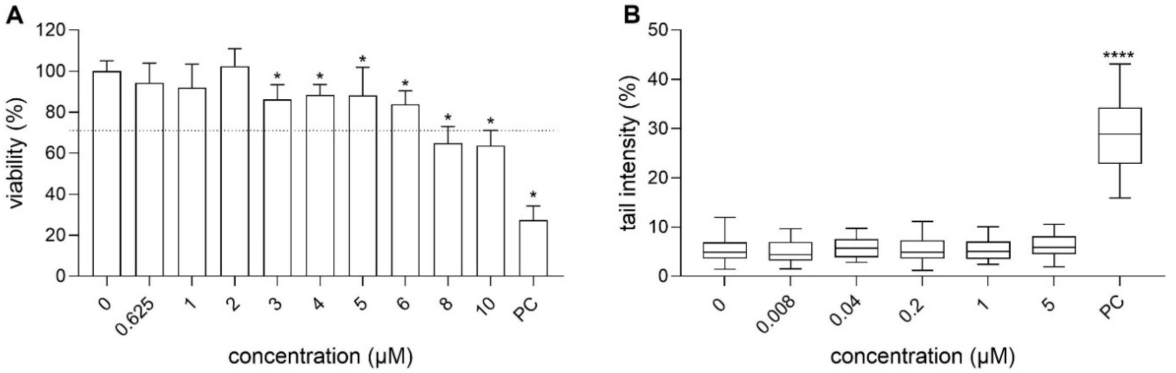

Figure S1. Cytotoxicity and genotoxicity of RA after 24 h exposure. (**A**) The effect of RA on the viability of HepG2 cells, presented as a percentage of the solvent control (0). Positive control (PC; DMSO 4%) was included in the experiment. The dashed line represents 70 % survival regarding to solvent control. * denotes a significant difference in comparison with 0 (* $p < 0.05$). (**B**) The effect of the RA on DNA damage induction. Data are expressed as % of DNA in the comet tail and presented as quantile box plots (95 % confidence interval) with the mean value in the form of a solid line through the box. BaP 30 μM was used as PC and 0 as solvent control. There were no differences between solvent control and growth medium control observed (data not shown). * denotes a statistically significant difference between the AFB1 and the exposed cells (Kruskal–Wallis nonparametric test and Dunn's multiple comparison test) (**** $p \leq 0.0001$).

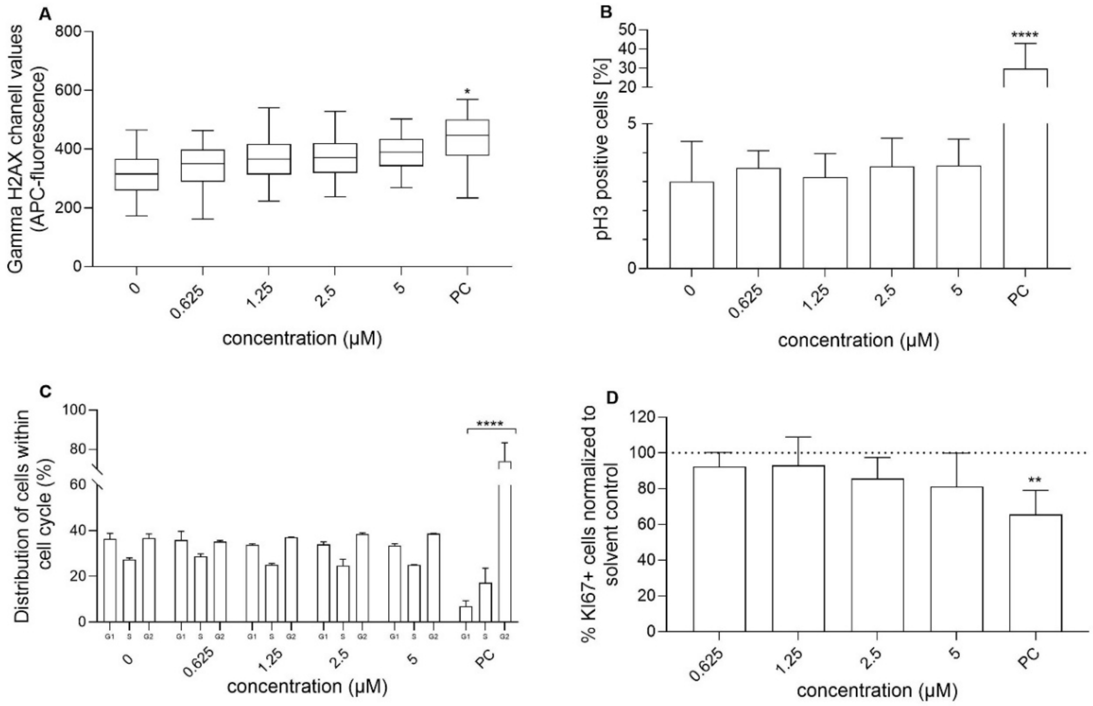

Figure S2. Potential of RA to induce DNA double strand breaks (**A**), alterations in the phosphorylation of the histone H3 (p-H3) (**B**), distribution of cells among the phases of the cell cycle (**C**), and the percentage of Ki67 positive cells (**D**) in HepG2 cells after 24 h exposure to RA of different concentrations. Solvent control (0) and positive control (PC; colchicine 0.1 μM for the phosphorylation of the p-H3 histone and etoposide 1 μg/mL for the remaining three plots) were included in the experiment. * denotes a significant difference in comparison with solvent control (* $p \leq 0.05$, ** $p \leq 0.01$, **** $p \leq 0.0001$).
